# Supplementary material for: Asynchronous magnetic resonance elastography: Shear wave speed reconstruction using noise correlation of incoherent waves
Source: Magn Reson Med. 2022 Oct 27;89(3):990–1001. doi: 10.1002/mrm.29502 (PMC9792433; doi:10.1002/mrm.29502)
Supplement: Supplementary file 1 — DATA S1 MATLAB code used to generate the numerical simulations of Figure 1 [file MRM-89-990-s002.zip › k-Wave/helpfiles/makeSphericalSection.html]

makeSphericalSection :: Functions (k-Wave)


# makeSphericalSection

Create a binary map of a sphere segment within a 3D grid.

## Syntax

```
ss = makeSphericalSection(radius, height)
ss = makeSphericalSection(radius, height, width)
ss = makeSphericalSection(radius, height, width, plot_section)
ss = makeSphericalSection(radius, height, [], plot_section)
ss = makeSphericalSection(radius, height, width, plot_section, binary)
ss = makeSphericalSection(radius, height, [], [] binary)

[ss, dist_map] = makeSphericalSection(radius, height)
[ss, dist_map] = makeSphericalSection(radius, height, width)
[ss, dist_map] = makeSphericalSection(radius, height, [], plot_section)
[ss, dist_map] = makeSphericalSection(radius, height, width, plot_section)
[ss, dist_map] = makeSphericalSection(radius, height, width, plot_section, binary)
[ss, dist_map] = makeSphericalSection(radius, height, [], [] binary)
```

## Description

`makeSphericalSection` creates a binary map of a section of a spherical surface within a three-dimensional matrix. The sphere is created using an extension of the midpoint circle algorithm. A single grid point is taken as the sphere center so the total diameter will always be an odd number of grid points. The sphere is then truncated based on the values for height and width (a diagram of the input sizes is given below). The face of the spherical section faces in the positive x-direction and the optional width parameter truncates the size in the y-direction.

If the optional input parameter `binary` is set to `false` (the default), the section map is returned as a double precision matrix. If it is set to `true`, the map is returned as a logical matrix. The average distance between each grid point in the spherical section and its contiguous neighbours can also be returned. This is given as a ratio compared to the average neighbour distance for a flat surface.

## Examples

```
makeSphericalSection(25, 6, 15, true);
```

```
makeSphericalSection(50, 20, [], true);
```

## Inputs

|  |  |
| --- | --- |
| `radius` | radius of curvature [grid points] |
| `height` | transducer height [grid points] |

## Optional Inputs

|  |  |
| --- | --- |
| `width` | section width (must be specified as an odd number) [grid points] |
| `plot_section` | Boolean controlling whether the spherical section is plotted using `voxelPlot` (default = `false`) |
| `binary` | Boolean controlling whether the spherical section is returned as a double precision matrix (`false`) or a logical matrix (`true`) (default = `false`) |

## Outputs

|  |  |
| --- | --- |
| `ss` | binary matrix containing spherical section |
| `dist_map` | ratio of average neighbour distance for each grid point within the spherical section compared to a flat surface |

## See Also

`makeBall`, `makeBowl`, `makeSphere`
